# Post-interventional Evaluation and Follow-Up in Children With Patent Ductus Arteriosus Complicated With Moderate to Severe Pulmonary Arterial Hypertension: A Retrospective Study

**DOI:** 10.3389/fcvm.2021.693414

**Published:** 2021-11-11

**Authors:** Xing Rong, Qiaofang Ye, Qiaoyu Wang, Jiajun Wang, Qiongjun Zhu, Youran Chen, Rongzhou Wu

**Affiliations:** Children's Heart Center, The Second Affiliated Hospital and Yuying Children's Hospital of Wenzhou Medical University, Institute of Cardiovascular Development and Translational Medicine, Wenzhou Medical University, Wenzhou, China

**Keywords:** patent ductus arteriosus, pulmonary arterial hypertension, transcatheter closure, evaluation, follow-up

## Abstract

**Background:** Transcatheter closure is an important treatment for patent ductus arteriosus (PDA) complicated with moderate and severe pulmonary arterial hypertension (PAH). This report presents our experience with transcatheter closure of PDA complicated with moderate and severe PAH.

**Methods:** The 49 cases of PDA complicated with moderate and severe PAH were collected in the Second Affiliated Hospital and Yuying Children's Hospital from January 2014 to December 2019 with transcatheter closure of PDA and follow-up. All patients were invited for transthoracic echocardiography, electrocardiogram, and thoracic radiography check-up.

**Results:** Device implantation was successful in 48 of 49 patients (98.0%). Among them, 30 cases were in the PAH after defect correction (CD) group, and 19 examples were in the Non-PAH after defect correction (NCD) group. Pulmonary systolic pressure, left atrial diameter, and left ventricular end-diastolic diameter immediately after interventional therapy and 6 months later were lower than the pre-operative levels (*p* < 0.05). The incidence of the immediate residual shunt (RS) in this study was 34.9%, most of which were minimal amount shunt. RS disappeared in all patients within 1 year of therapy. Four patients had thrombocytopenia and one patient had left pulmonary artery stenosis. No other serious adverse event occurred during the follow-up period. The pressure gradient tricuspid valve regurgitation (PGTI) and the right heart catheterization (RHC) consistency points were 93.75% (15/16) and were within the 95% consistency limit by the Bland-Altman method. The Logistic regression analysis concluded that the pre-operative Pp/Ps and the narrowest diameter of PDA are risk factors for post-operative PAH (*p* < 0.05). The cut-off point of the pre-operative Pp/Ps and the narrowest diameter of PDA were calculated to be 0.595 and 4.75 mm, respectively.

**Conclusion:** Interventional occlusion in children with PDA complicated with moderate and severe PAH is safe, effective, and has few complications. Targeted drug therapy has a good clinical effect. The narrowest diameter of PDA and the pre-operative Pp/Ps may be one of the risk factors of residual PAH after interventional therapy.

## Introduction

Patent ductus arteriosus is one of the most common congenital heart diseases (CHD) (1). One of its common and serious complications is pulmonary arterial hypertension (PAH). In the presence of patent ductus arteriosus (PDA), many long-term left-to-right shunts directly wash out the pulmonary artery and are easy to cause PAH in the early stage. Children with PDA complicated with moderate and severe PAH can be treated by interventional occlusion or surgery. Because of less trauma, rapid recovery after interventional closure, observation of pulmonary artery pressure, and the advantages of recyclable occluder during operation, interventional occlusion has become the first-choice treatment for these children (2, 3). Due to the vast territory and the uneven economic development of various regions in China, many CHDs are not detected in time and progress to moderate and severe PAH. If the patients are not treated in time, they will develop Eisenmenger's syndrome (ES) and lose the best chance of treatment (4). For patients with PDA combined with moderate to severe PAH, intraoperative trial closure and post-operative evaluation are the advantages of interventional therapy. The prognosis of these patients is still unclear. Although the ratio of pulmonary to the systemic circulation (Qp/Qs) > 1.5, some patients still have different degrees of PAH after occlusion. To our knowledge, there are few reports about the results of patients with PAH after occlusion. The purpose of this study is to evaluate the effectiveness, safety, and medium- and short-term prognosis of interventional occlusion in children with PDA complicated with moderate and severe PAH.

## Methods

### Selection of Patients

The 49 cases of PDA complicated with moderate and severe PAH with successfully transcatheter closure of PDA and discharged with follow-up were collected in the Second Affiliated Hospital and Yuying Children's Hospital of Wenzhou Medical University from January 2014 to December 2019. After admission, all children were given a comprehensive clinical assessment, including medical history, physical signs, laboratory examination, and auxiliary examination. The following inclusion criteria were applied (5): (1) Mean pulmonary artery pressure (mPAP) > 41 mmHg or pulmonary arterial systolic pressure (PASP) > 46 mmHg measured by catheterization; (2) Qp/Qs > 1.5; and (3) Left ventricular ejection fraction (LVEF) > 50% by pre-operative echocardiography. The exclusion criteria were patients who needed surgery at the same time. This study was approved by the Ethics Committee of the Second Affiliated Hospital of Wenzhou Medical University. Informed consent was obtained from each patient's parents or themselves.

### Procedure

The study was approved by the local institutional ethics committee. Informed consent was obtained from all patients and/or their parents. All children underwent general anesthesia. The heart rate, heart rhythm, and pulse oxygen saturation were continuously monitored during the procedure. Routine right femoral arteriovenous catheterization was performed to measure the pulmonary capillary wedge pressure and pressures at each cardiac chamber and big artery. Blood gas analysis was performed at each site. Oxygen consumption was estimated from the age and body surface area of the patient. Cardiac output was measured with the Fick method, and the Qp/Qs, post-void residual (PVR), and systemic vascular resistance were calculated with standard formulae. Routine angiography of the lateral and right anterior oblique position descending aorta was done to determine the location, size, type, and whether the PDA is combined with aortic coarctation and other deformities. According to the results of angiography, the appropriate PDA occluder was selected to seal the PDA. The device was placed into the PDA while still attached to the cable. Saline-filled catheters continuously measured the pulmonary artery pressure (PAP) and apical pulse (AP) before the device was released. Occluder release met the following conditions: (1) After blocking PAP decreased by 30% or more, or if the PAP or closure mPAP reduced by 20% or more 30 mmHg, while the AP and SaO_2_ without falling or rising; (2) no residual shunt or only a small shunt remained; (3) there was no obvious abnormality in vital signs.

### Follow-Up

Patients underwent serial follow-up examinations at 24 h, 1, 3, 6, and 12 months, and then yearly after the procedure.

### Monitoring of Pulmonary Hypertension

#### Gold Standard

Right heart catheterization (RHC): the ratio of pulmonary artery pressure to aortic pressure (Pp/Ps) can also be used as a classification standard wherein the ratio <0.45 is mild PAH, the ratio of 0.45–0.75 is moderate PAH, and the ratio > 0.75 is severe PAH (6).

#### Ultrasound Cardiogram Estimation of PAH

The tricuspid regurgitation peak velocity (TRV) was measured by continuous-wave Doppler echocardiography. The peak pressure gradient between the right ventricle and right atrium was calculated with the simplified Bernoulli equation, as 4v^2^ plus right atrial pressure (5 mmHg), and if the inferior vena cava is dilated, the right atrium pressure is 10 mmHg.

#### Electrocardiogram Estimation of PAH

Right ventricular hypertrophy (RVH) diagnostic criteria: V1 R/S > 1.0, V5 R/S <1.0, RV1 + SV5 > 1.05 mV, R aVR > 0.5 mV, R V1 > 1.0 mV, QRS axis > +110°.

### Statistical Analysis

Statistical analysis using SPSS 23.00 statistical software. Continuous data were expressed as means ± SDs with ranges and were compared using ANOVA and the Student's *t*-test, while categorical data were expressed as number and percentage, and were compared using the χ^2^-test. Risk factor analysis using multivariate Logistic regression analysis. *P-*values <0.05 were considered to be significant.

## Results

### General Information

Forty-nine children with PDA complicated with middle and severe PAH were included in this study. Among them, 48 cases had a successful intervention, and 1 case gave up. The success rate of occlusion can reach 98%, which is higher than previous reports. The 48 children were divided into two groups according to the presence or absence of PAH after the operation. Characteristics of the study population are shown in Table 1. There was no significant difference in general indexes between the two groups (*p* > 0.05), indicating that the data of the two groups were comparable (Table 1).

**Table 1:** General characteristics of patients.

	**CD group**	**NCD group**	***P***
Case, *n* (%)	30 (63%)	18 (37%)	-
Female sex, *n* (%)	19 (67%)	14 (78%)	0.402
Length of hospitalization (d)	9.98 ± 6.49	10.95 ± 6.50	0.619
Age at procedure (m)	11.50 (6.75, 27.00)	12.00 (5.75, 30.75)	0.553
Height at procedure (cm)	74.00 ± 15.02	76.91 ± 21.46	0.583
BSA(m^2^)	0.40 ± 1.31	0.42 ± 2.44	0.576
**Cardiac function**, ***n*** **(%)**			
Normal	22 (73%)	10 (56%)	-
Mild	6 (20%)	5 (28%)	-
Moderate	2 (7%)	3 (17%)	-
Severe	0	0	-
Recurrent respiratory infections, *n* (%)	3 (10%)	3 (17%)	0.822
NT-proBNP (ng/dl)	913.5 (505.5, 1,690)	905.0 (457.3, 1,637.5)	0.499
CTR (%)	58.41 ± 11.95	59.36 ± 3.16	0.745
LVEF (%)	70.93 ± 6.14	70.67 ± 6.03	0.884
Pre-operative cardiac medications *n* (%)	4 (13%)	3 (17%)	1.000

*BSA, body surface area; NT-proBNP, N terminal pro B type natriuretic peptide; CTR, cardiothoracic ratio; LVEF, left ventricular ejection fraction*.

### Intraoperative Data

Intraoperative angiography showed that the PDA form differed. Particularly, there were 21 cases of funnel type, one case of window type, and eight cases of tube type in the PAH after defect correction (CD) group. In the Non-PAH after defect correction (NCD) group, 12 PDA cases were funnel type, five were tube type, and one was atypical. The Qp/Qs of the CD group was 1.95 ± 0.59, the PVR was 7.05 ± 1.20 Wu, and the average operation time was 1.88 ± 0.56 h. The Qp/Qs of the NCD group was 2.21 ± 0.78, the PVR was 6.79 ± 2.10 Wu, and the average operation time was 1.85 ± 0.60 h. There was no statistical difference in the above indicators between the two groups (*p* > 0.05). The average PDA size of the CD group was 5.20 ± 1.55 mm, and the average pre-operative Pp/Ps was 0.70 ± 0.12. The average PDA size of the NCD group was 4.05 ± 0.63 mm, and the pre-operative Pp/Ps of the NCD group was 0.60 ± 0.09. There was a statistically significant difference in the above indicators between the two groups (*p* < 0.05). The PASP immediately after the operation in both groups decreased by more than 20% compared with before, and the difference was statistically significant (*p* < 0.05). There was no difference between aortic pressure immediately after surgery and pre-operatively (*p* > 0.05; Table 2).

**Table 2:** Clinical data of CD group and NCD group.

	**CD group**	**NCD group**	***P***
Procedure time (h)	1.88 ± 0.56	1.85 ± 0.60	0.860
Radiation time (h)	0.74 ± 0.12	0.677 ± 0.32	0.898
PDA size (mm)	5.20 ± 1.55	4.05 ± 0.63	0.006*
Pulmonary artery diameter of occluder (mm)	10.30 ± 4.27	8.56 ± 1.15	0.042^*^
Aortic diameter of occluder (mm)	13.03 ± 4.41	11.22 ± 2.29	0.065
**PDA type**, ***n*** **(%)**
Type A	21 (70%)	12 (67%)	
Type B	1 (3%)	0	
Type C	8 (27%)	5 (28%)	
Type D	0	0	
Type E	0	1 (5%)	
Qp/Qs	1.95 ± 0.59	2.21 ± 0.78	0.447
PVR (Wu)	7.05 ± 1.20	6.79 ± 2.10	0.258
Pre-operative Pp/Ps	0.70 ± 0.12	0.60 ± 0.09	0.001^*^
Moderate PAH;0.45–0.75	*N* =20	*N* = 15	
Severe PAH; >0.75	*N* =10	*N* = 3	
Post-operative Pp/Ps	0.46 ± 0.13	0.27 ± 0.05	0.006^*^
Mild PAH; <0.45	*N* = 17	0	
Moderate PAH;0.45–0.75	*N* = 13	0	
Pre-operative PASP (mmHg)	60.13 ± 10.83	50.17 ± 7.90	0.001^*^
The change range of PASP before and after operative (%)	0.34 ± 0.10	0.50 ± 0.09	
DAO pre-operative (mmHg)	88.60 ± 16.54	88.11 ± 11.74	0.906
DAO post-operative (mmHg)	90.40 ± 16.26	91.50 ± 14.46	0.809

*Qp/Qs, pulmonary to systemic flow ratio; PVR, pulmonary vascular resistance; Pp/Ps, pulmonary to systemic pressure ratio; PASP, pulmonary arterial systolic pressure; DAO, descending aorta*.

* *p-value of <0.05*.

### Post-operative Data

#### Echocardiographic Data

After 1, 3, and 6-month follow-up, the left atrial diameter (LAD), left ventricular end-systolic diameter (LVESD), and left ventricular end-diastolic diameter (LVEDD) decreased significantly (*p* < 0.05). There was no progressive reduction in the above-mentioned indexes in the two groups during the 1 year after the operation. During the first follow-up to 3 months after the procedure, the ejection fraction (EF) in the two groups decreased significantly (*p* < 0.05), but there was no progressive reduction in 6 months follow-up (Table 3).

**Table 3:** Echocardiography data of CD group and NCD group during pre-operative and post-operative periods.

**Group**	**Parameter**	**Pre-operative**	**3 d**	**1 m**	**3 m**	**6 m**	**1 y**	**2 y**
CD group	LVESD	21.57 ± 5.13	21.03 ± 4.95^*^	19.03 ± 4.19^*^	18.22 ± 3.36^*^	18.61 ± 3.67^*^	18.50 ± 2.76	19.73 ± 2.90
	LVEDD	35.20 ± 7.14	32.13 ± 6.04^*^	29.96 ± 5.10^*^	29.51 ± 4.13^*^	29.00 ± 3.73^*^	30.34 ± 3.36	31.71 ± 2.83
	LAD	25.63 ± 5.64	22.6 ± 4.47^*^	21.21 ± 4.45^*^	21.03 ± 4.25^*^	21.42 ± 4.18^*^	23.08 ± 3.38	24.23 ± 3.21
	LVEF	70.93 ± 6.14	63.10 ± 14.34^*^	66.35 ± 14.78^*^	69.31 ± 7.67^*^	69.88 ± 5.36	70.46 ± 4.96	69.52 ± 6.27
NCD group	LVESD	22.11 ± 4.39	20.72 ± 4.32^*^	19.18 ± 6.65^*^	17.71 ± 4.36^*^	16.45 ± 3.01^*^	17.90 ± 2.13	18.50 ± 0.60
	LVEDD	36.83 ± 6.68	31.50 ± 5.87^*^	27.88 ± 3.44^*^	28.92 ± 6.09^*^	27.84 ± 4.39^*^	28.40 ± 2.59	30.89 ± 3.18
	LAD	25.11 ± 4.33	21.27 ± 3.41^*^	19.43 ± 3.44^*^	20.36 ± 2.84^*^	19.09 ± 3.53^*^	20.77 ± 4.58	20.57 ± 8.72
	LVEF	70.67 ± 6.03	64.22 ± 6.58^*^	67.56 ± 6.51^*^	69.77 ± 3.38^*^	70.81 ± 5.79	71.08 ± 5.12	73.00 ± 3.21

*LVESD, left ventricular end systolic diameter; LVEDD, left ventricular end diastolic diameter; LAD, left atrial diameter; LVEF, left ventricular ejection fraction*.

**#x0002A;:** *p-value of <0.05 vs. pre-operative data*.

#### Chest Radiography Data

The cardiothoracic ratio (CTR) of the two groups at 1, 3, and 6 months after the operation was significantly smaller than that before operation (*p* < 0.05), suggesting that the cardiac size was smaller after the operation pre-operatively (Table 4).

**Table 4:** Cardiothoracic ratio during pre-operative and post-operative periods.

	**Cardiothoracic ratio (%)**
Pre-operative	59.80 ± 3.75
1 m	56.19 ± 4.43^*^
3 m	55.46 ± 4.50^*^
6 m	55.63 ± 5.96^*^

* *p-value of <0.05 vs. pre-operative data*.

### Treatment of Post-operative Residual PAH

**Figure 1:**
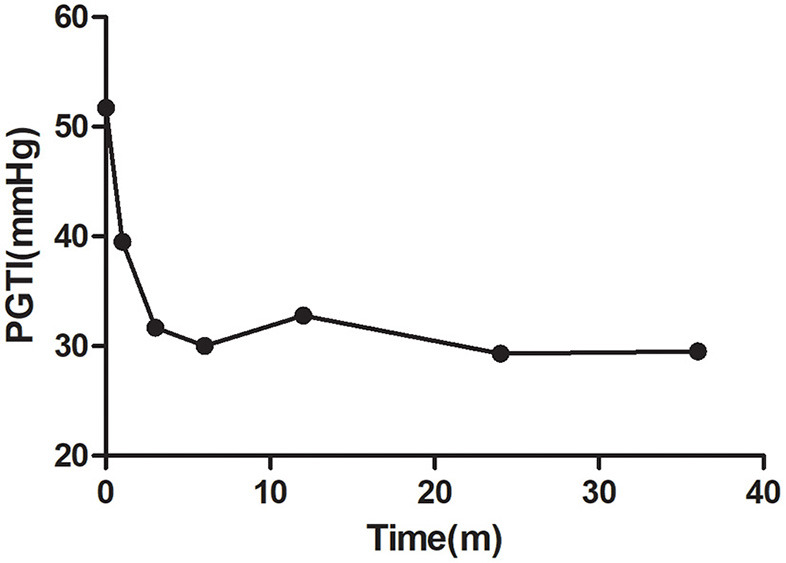
Changes of pressure gradient tricuspid valve regurgitation (PGTI) in patients with oral Bosentan after the operation.

**Figure 2:**
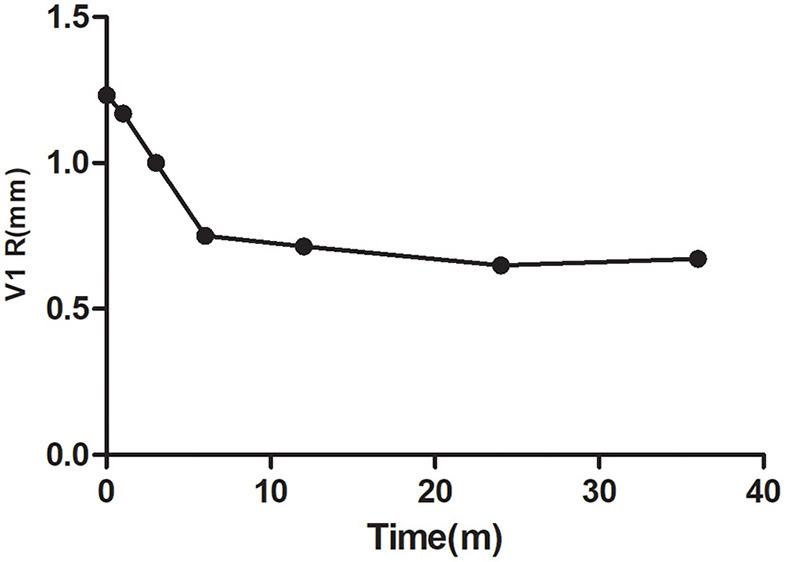
Changes of V1 R in patients with oral Bosentan after the operation.

In the CD group, 17 cases were complicated with mild PAH, and 13 cases had moderate PAH after defect correction. According to the pulmonary to systemic pressure ratio before operation and age, 18 children were treated with Bosentan after the operation. Among them, 14 children had tricuspid regurgitation. The pressure gradient tricuspid valve regurgitation (PGTI) decreased over time after interventional occlusion (*p* < 0.05; Figure 1). In 18 cases, the R wave amplitude of V1 decreased significantly over time after interventional occlusion (*p* < 0.05; Figure 2).

After taking Bosentan for 1 month, abnormal liver function was found in two children. Their alanine aminotransferase (ALT) increased by more than three times the normal value. The liver function returned to normal after stopping Bosentan treatment and giving hepatoprotective treatment. The PAH in these two cases returned to normal during follow-up.

### Monitoring of PAH

**Figure 3:**
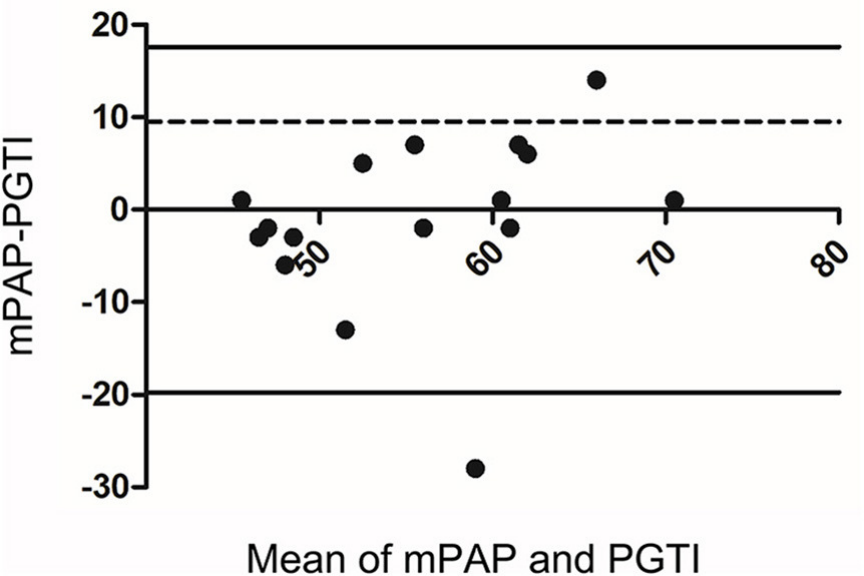
Bland-Altman analysis of pulmonary arterial systolic pressure (PASP) between cardiac catheterization and estimation of tricuspid regurgitation.

**Figure 4:**
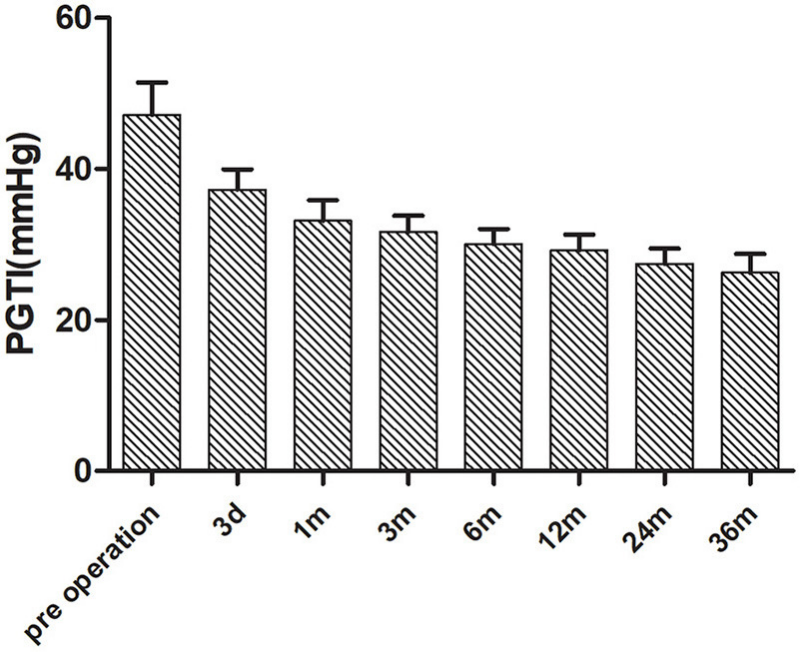
Changes of PGTI in patients with pre-operative tricuspid regurgitation after the operation.

Post-operative follow-up was performed by echocardiogram, electrocardiogram, and RHC. Only 16 cases (53%) in the CD group had tricuspid regurgitation before the operation. The Bland-Altman method was used to analyze the consistency between the two methods of PASP by measuring the PGTI and the RHC in which it was found that consistency points were 93.75% (15/16) and were within the 95% consistency limit, which showed good consistency (Figure 3). The PASP measured by PGTI of the 3 days, 1, 3, 6, 12, 24, and 36 months of the post-operative follow-up was lower than the previous one. The difference is statistically significant (*p* < 0.05; Figure 4).

Pearson correlation analysis (Table 5) concluded that Rv1, Rv1 + Sv5, QRS axis, and V1R/S on ECG are positively correlated with mPAP monitored by RHC, and the correlation between Rv1 and V1R/S is higher.

**Table 5:** Correlation of mean pulmonary arterial pressure (mPAP) with parameters of ECG in patients during the post-operative period.

**Variables**	**mPAP**	
	***r***	***P***
Rv1	0.390	0.001^*^
Rv5	−0.266	0.039
Rv1 + Sv5	0.320	0.011^*^
QRS axis	0.289	0.006^*^
Sv5	0.290	0.056
V1 R/S	0.550	0.001^*^

*mPAP, mean pulmonary artery pressure*.

* *p-value of <0.05*.

One year after the intervention, nine children underwent right heart catheterization again. The values of PASP, mPAP, and Pp/Ps were 30.21 ± 9.30 mmHg, 45.50 ± 6.93 mmHg, and 0.74 ± 0.14, respectively, which were all lower than the immediate values after intervention (Table 6).

**Table 6:** Post-operative follow-up of related indexes of pulmonary arterial hypertension after the operation.

**Variables**	**First time**	**Second time**	***P***
PASP (mmHg)	61.62 ± 8.48	36.50 ± 13.14	<0.001
mPAP (mmHg)	45.50. ± 6.93	23.37 ± 5.04	<0.001
Pp/Ps	0.74 ± 0.14	0.41 ± 0.15	<0.001

*PASP, pulmonary arterial systolic pressure; mPAP, mean pulmonary artery pressure; Pp/Ps, pulmonary to systemic pressure ratio*.

### Analysis of Risk Factors for PAH Still Existing After the Operation

Logistic regression analysis concluded that the pre-operative Pp/Ps and the narrowest diameter of PDA were risk factors for post-operative PAH (*p* < 0.05; Table 7).

**Table 7:** Logistic regression analysis on risk factors of CD group.

**Risk factors**	**Regression coefficient**	**Standard error**	***P***	**OR**	**95% CI**
PDA size	1.836	0.928	0.048^*^	6.274	1.017–38.720
Pre-operative Pp/Ps	0.086	0.042	0.042^*^	1.089	1.003–1.183
PA diameter of occluder (mm)	−0.041	0.267	0.879	0.960	0.568–1.622

*PDA, patent ductus arteriosus; PA, patent artery*.

* *p-value of <0.05*.

**Figure 5:**
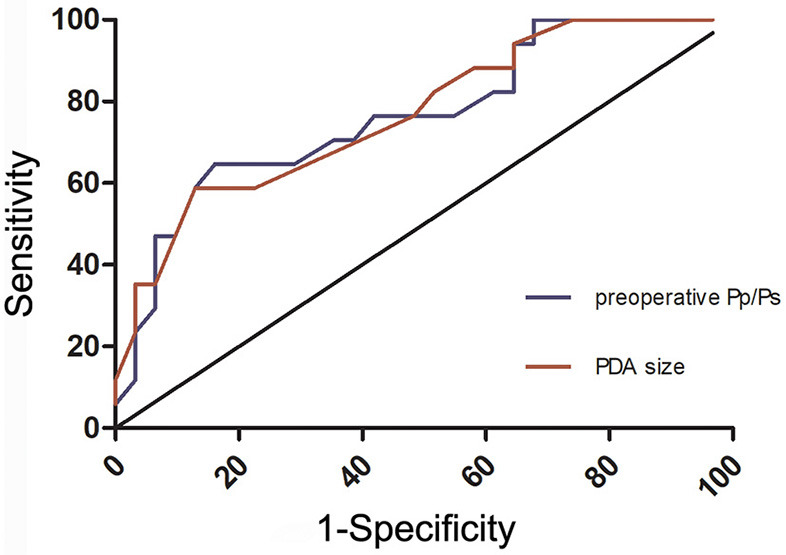
Receiver operating characteristic (ROC) curve of patent ductus arteriosus (PDA) size and the pre-operative ratio of pulmonary artery pressure to aortic pressure (Pp/Ps) between CD and NCD groups.

The receiver operating characteristic (ROC) curve was used to calculate the critical value of variables, that is, the value of each variable when the Youden index is the largest. The cut-off point of the pre-operative Pp/Ps and the narrowest diameter of PDA are calculated to be 0.595 and 4.75 mm, respectively (Figure 5).

## Discussion

Patent ductus arteriosis is one of the most common congenital heart diseases (1). One of its most common and serious complications is PAH. In the presence of PDA, many long-term left-to-right shunts directly wash out the pulmonary artery and are easy to cause PAH in the early stage. Children with PDA complicated with moderate and severe PAH can be treated by interventional occlusion or surgery. Because of less trauma, rapid recovery after interventional closure, observation of pulmonary artery pressure, and the advantages of recyclable occluder during operation, interventional occlusion has become the first choice for these children (2, 3). Due to the vast territory and the uneven economic development of various regions in China, many CHDs are not detected in time and progress to moderate and severe PAH. If the patients are not treated in time, they will develop ES and lose the best chance of treatment. For patients with PDA combined with moderate to severe PAH, intraoperative trial closure and post-operative evaluation are the advantages of interventional therapy. The prognosis of these patients is still unclear. Although pre-operative Qp/Qs > 1.5, some patients still have different degrees of PAH after occlusion. To our knowledge, there are few reports about the results of patients with PAH after occlusion. The purpose of this study is to evaluate the effectiveness, safety, and medium and short-term prognosis of interventional occlusion in children with PDA complicated with moderate and severe PAH.

### Effectiveness Analysis

For patients complicated with moderate to severe PAH, the occlusion test can be used during intervention to determine the degree of pulmonary vascular disease and the changes in PAH after surgery. In this study, the success rate of interventional blockade of PDA combined with moderate to severe PAH was 98%, which was higher than previous reports (7). The post-operative PAP of all patients was reduced by more than 20% compared with that before the closure, the AP and SaO_2_ did not decrease, and there was no systemic response.

Post-operative residual shunt (RS) is the most important indicator for evaluating the curative effect of interventional therapy. The incidence of immediate RS in this study was 34.9%, most of which were minimal shunts. RS disappeared in all patients within 1 year after therapy, which was consistent with previous reports (8). Therefore, we think that most of this mild RS can disappear after the operation and does not need special treatment.

Furthermore, the LAD, LVEDD, and LVESD of all infants decreased by the 6-month follow-up and eventually returned to normal, consistent with the results of a previous study (9).

Therefore, interventional closure of PDA with moderate to severe PAH has a definite effect.

### Security Analysis

Four patients in the CD group had thrombocytopenia after surgery. The mechanism of that is not fully clear. Some scholars believe that it is related to excessive platelet consumption or destruction (10). One case had left pulmonary artery (LPA) stenosis during the 1-year follow-up. After completing RHC and cardiac CT angiography (CTA), we found that the LPA was slender and blood flow was still visible. This can provide an opportunity for this child to receive a stent in the future. A close follow-up will be carried out for this case. Other complications such as descending aortic stenosis, displacement or shedding of the occluder, mechanical hemolysis, infective endocarditis, and thromboembolism did not occur during the follow-up process. Interventional closure of PDA with moderate to severe PAH has few complications and high safety.

### Post-operative Evaluation of PAH

This study showed that in children with PDA with moderate to severe PAH and Qp/Qs > 1.5, although PAP decreased significantly post-operatively, only 39.5% of children with PAP could return to normal immediately, while 23% of children had moderate PAH, and 40% of the children had mild PAH after the intervention. In the CD group, 18 children were given Bosentan post-operative. During the follow-up process, 29% of patients still had PAH until 3 months after therapy, 4.2% of patients had PAH persisting after therapy.

Different from other types of PAH, PAH caused by CHD includes two types: correctable and uncorrectable. In the early stage, PAH relies on the left to right shunting, and closing the defect at this time can make PAP return to normal post-operatively. As PAP continues to rise, pulmonary vascular remodeling gradually causes irreversible lesions (11). At this time, PAH no longer depends on the left to right shunting. Closing the defect does not reduce PAP to normal at this stage. On the contrary, due to the loss of the buffering effect of the defect, it can cause a shortening of life. The prognosis of these patients is similar to idiopathic PAH. There is no unified standard to distinguish between dynamic and resistance PAH in severe PAH without ES. Early research indicated that lung biopsy could be used as the gold standard (12). However, subsequent studies found that lung biopsy is of limited value in judging the prognosis of children with CHD (13, 14). The reason is that the pulmonary vascular disease caused by CHD is not uniformly distributed, and it is difficult to predict the severity of overall pulmonary vascular disease with local tissue disease. At present, the clinical gold standard for identifying whether CHD complicated with PAH has surgical indications is the RHC. Although the data of the RHC has important guiding significance for whether there are surgical indications, there are no clear indicators and standards to thoroughly distinguish the nature of PAH. Because of this, the operator must determine whether the patient has surgical indications based on the clinical manifestations of the patient and his own experience. The 2010 European Society of Cardiology (ESC) adult CHD management guidelines still use Qp/Qs > 1.5 as the criterion for distinguishing dynamic and resistance PAH (15). PAP increased significantly and the index of Qp/Qs <1.5 indicated that the patient has entered the resistance PAH stage. A significantly increased PAP and the index of Qp/Qs <1.5 indicate that the patient has entered the resistance PAH stage. Our study shows that in children with PDA and moderate to severe PAH, only 39.5% can return to normal immediately after surgery. In this study, 18 children were given Bosentan targeted drug therapy after surgery. Only two patients suffered liver damage 1 month after taking the medication. The other Bosentan-related adverse reactions, such as facial flushing, were not present. The liver function returned to normal after the drug was stopped and hepatoprotective treatment was initiated. During follow-up, the degree of PAH gradually recovered. By 3-months follow-up after the operation, there were 29% of patients still having PAH. PAH persisted in 4.2% of patients.

Several domestic and foreign studies have shown that targeted drugs to treat PAH can improve patient hemodynamics, exercise tolerance, quality of life, and survival (16, 17). At present, the treatment of CHD-PAH patients in China is mainly focused on the pre-operative and perioperative periods (18, 19). Most of the patients failed to adhere to targeted drugs and regular follow-up after surgical treatment. The results of this study show that patients need early targeted drug interventions with moderate to severe PAH after surgery, and the incidence of long-term adverse events is low. For children with residual PAH after closure, the treatment of PAH should not be limited to the perioperative period and a short period of time after the operation. More importantly, the long-term targeted drug therapy and regular follow-up after the operation are safe and effective.

Right heart catheterization is the gold standard for diagnosing PAH, but it is an invasive and expensive test. It is difficult to be popularized in the follow-up of children with PAH after surgery. In this study, only 10 children (33%) were admitted to the hospital for right heart catheterization after occlusion for 1–2 years. The calculation of PASP by continuous Doppler ultrasound measurement of the peak tricuspid regurgitation velocity is non-invasive, easy to operate, and reproducible. It has always been the main method for PAH screening. The PASP and right heart catheter pressures estimated by the tricuspid regurgitation method demonstrate good consistency. A large number of studies have confirmed this conclusion (20). The study also shows that the difference in PASP obtained by the two methods is not statistically significant and has a good consistency, which can be used to assess post-operative PAH. However, not all children with PDA and PAH have tricuspid regurgitation. In this study, only 15 children in the CD group had tricuspid regurgitation before surgery. The remaining 15 children without tricuspid regurgitation encountered a bottleneck in the follow-up of PAH after surgery. Studies have shown that ECG has become an important examination item in the diagnosis process of PAH. In 2008, Al-Naamani et al. (21) reported the results of a clinical study on the diagnostic value of 12-lead electrocardiography for PAH. The results found that the amplitude of R and S and R/S ratio in lead V1 has the greatest predictive value for PAH. The results of this study are similar to previous studies, indicating that the lead V1 R wave amplitude and R/S ratio in lead V1 have important clinical value in the assessment of PAH.

Although pre-operative Qp/Qs > 1.5 and intraoperative occlusion test is normal, it is difficult for some children to avoid post-operative residual PAH. In this study, we further discussed the risk factors of post-operative residual PAH in children with moderate and severe PAH after interventional occlusion. This study is the first to clarify that pre-operative Pp/Ps and the narrowest diameter of PDA are risk factors for residual PAH after the intervention. In clinical work, the patients with pre-operative Pp/Ps > 0.595 or the narrowest diameter of PDA > 4.75 mm are more likely to retain some PAH after the operation. Clinicians should pay more attention to grasp the surgical indications and intervene in time to avoid irreversible changes in pulmonary vessels and lose the opportunity of operation.

## Conclusion

Interventional occlusion in children with PDA complicated with moderate and severe PAH is safe, effective, and has few complications. Targeted drug therapy has a good clinical effect. The narrowest diameter of PDA and the pre-operative Pp/Ps may be risk factors of residual PAH after interventional therapy.

## Limitation

The shortcomings of this study are the use of UCG and ECG to estimate PAP in the follow-up after the operation, not all *via* RHC. After the operation, only 10 children were further evaluated with RHC to determine whether PAH remained. In addition, this study is a retrospective study with limitations, and further prospective, randomized, large-scale, long-term studies are needed to validate our findings.

## Data Availability Statement

The raw data supporting the conclusions of this article will be made available by the authors, without undue reservation.

## Ethics Statement

The studies involving human participants were reviewed and approved by Ethics Committee of the Second Affiliated Hospital of Wenzhou Medical University. Written informed consent to participate in this study was provided by the participants' legal guardian/next of kin.

## Author Contributions

RW conceived the study. XR and QY wrote the manuscript. QY analyzed the results. QW, JW, QZ, and YC collected the clinical data. All authors contributed to the article and approved the submitted version.

## Conflict of Interest

The authors declare that the research was conducted in the absence of any commercial or financial relationships that could be construed as a potential conflict of interest.

## Publisher's Note

All claims expressed in this article are solely those of the authors and do not necessarily represent those of their affiliated organizations, or those of the publisher, the editors and the reviewers. Any product that may be evaluated in this article, or claim that may be made by its manufacturer, is not guaranteed or endorsed by the publisher.
